# Regional differences in treatment and outcome for myeloma patients in Sweden: A population based Swedish myeloma register study

**DOI:** 10.1002/cnr2.1614

**Published:** 2022-03-03

**Authors:** Göran Wålinder, Anna Genell, Gunnar Juliusson, Ronald Svensson, Antonio Izarra Santamaria, Jacob Crafoord, Kristina Carlson, Dorota Knut‐Bojanowska, Ljupco Veskovski, Birgitta Lauri, Johan Lund, Ingemar Turesson, Markus Hansson, Cecilie Hveding Blimark, Hareth Nahi

**Affiliations:** ^1^ Department of Medicine Huddinge Karolinska Institute Solna Sweden; ^2^ Department of Hematology Karolinska University Hospital Huddinge Huddinge Sweden; ^3^ Regional Cancer Centre West Gothenburg Sweden; ^4^ Department of Hematology Lund University Lund Sweden; ^5^ Department of Hematology Linköping University Hospital Linköping Sweden; ^6^ Department of Hematology Umeå University Hospital Umeå Sweden; ^7^ Department of Hematology Örebro University Hospital Örebro Sweden; ^8^ Department of Hematology Uppsala University Hospital Uppsala Sweden; ^9^ Department of Haematology Uddevalla Hospital Uddevalla Sweden; ^10^ Department of Hematology Södra Älvsborgs Hospital Borås Sweden; ^11^ Department of Hematology Sundeby Hospital Luleå Sweden; ^12^ Department of Hematology Skane University Hospital Malmö Sweden; ^13^ Department of Hematology Sahlgrenska University Hospital Gothenburg Sweden

**Keywords:** cancer care, epidemiology, multiple myeloma, treatment outcome

## Abstract

**Background:**

We wanted to evaluate if health care for multiple myeloma (MM) patients is equal in different regions of Sweden.

**Aim:**

To study differences in survival for MM depending on health care region and early use of modern treatment.

**Methods and results:**

Data from the Swedish Myeloma Register from patients diagnosed between 2008 and 2017 was used. Cohorts were defined by the six healthcare regions (labeled A–F) in Sweden and modern initial treatment was defined as including certain drug combinations. To adjust for time to treatment bias, survival analyses were performed also for patients alive 6 months after diagnosis. In all treated MM patients (*n* = 5326), we observed a superior overall survival (OS) for region A compared to all other regions (*p* < .01 for all respectively). After adjusting for time to treatment there was also a superior survival in the region with highest use of modern initial treatment (region A) compared to the regions defined in the study as having intermediate and low use (*p* < .01 for both). In patients receiving autologous stem cell transplantation (ASCT) a superior survival was observed for region A compared to all regions besides region B. Similar results were seen when adjusting for a time to treatment bias. In patients not receiving ASCT, 75 years or older and adjusted for time to treatment bias, a difference was noted only between region A and E (log rank *p* = .04, HR 1.2, CI 1.00–1.44, *p* = .06). In multivariate analyses including age, international staging system stage and time period of diagnosis, differences in survival remained for patients receiving ASCT between region A versus C, D, E and F (*p* = .01, *p* < .01, *p* < .01, *p* = .03).

**Conclusion:**

We observed a superior survival in region A for patients receiving ASCT. Explanations may be higher usage of modern initial treatment or regional residual confounding. For patients not receiving ASCT, 75 years or older, differences in survival could be adjusted for.

## 1 INTRODUCTION

multiple myeloma (MM) is the second most common hematological malignancy.^1^ It is characterized by a steep increase in incidence with advancing age. In Sweden the crude annual incidence is 7/100 000 inhabitants and the median age at diagnosis has been estimated to be 70 years for men and 73 years for women.^2^

The survival for MM patients has increased since the introduction of high dose melphalan with autologous stem cell transplantation (ASCT) as part of standard treatment for younger patients.^3^, ^4^, ^5^ The introduction of proteasome inhibitors (PI: s),^6^, ^7^, ^8^, ^9^ immunomodulators (IMIDS: s)^10^, ^11^, ^12^, ^13^, ^14^, ^15^ and the combinations with PI: s and IMIDS: s^16^, ^17^ have led to gradual improvement of outcome over time, as has also the use of the anti‐CD38 monoclonal antibodies.^18^ Maintenance treatment with PI: s and IMID: s has also further contributed to better outcome.^19^

While improved survival has been demonstrated mainly in randomized clinical trials not including most myeloma patients, a dramatic improvement has been reported also in population based studies.^20^, ^21^, ^22^

Data from both the Swedish Lymphoma and the Swedish Leukemia Registries have demonstrated differences in outcome depending on which healthcare region in Sweden patients belong to.^23^, ^24^ In myeloma, treatment inequalities in subgroups of the population in other countries have also been earlier described.^25^, ^26^ Although there are national consensus guidelines in Sweden for treatment of lymphoma, leukemia and myeloma worked out by representatives from each health care region, the implementation and interpretation of these guidelines may still vary. As the goal is to provide an equal health care in the whole country, with all suitable treatment considered for all MM patients regardless of region and age, an evaluation of survival and treatment choice between the healthcare regions was considered necessary.

The aim of this study was to investigate possible differences in overall survival (OS) between healthcare regions in Sweden in relation to treatment with considerations to known confounding factors.

## 2 METHODS

Patients diagnosed with symptomatic MM from 2008‐01‐01 to 2017‐12‐31 registered in the population based Swedish Myeloma Register^27^, ^28^ were included in the study, provided that they also had a 1 year follow up report. The definition of symptomatic MM in the register used international myeloma working group (IMWG) criteria at the time.^29^, ^30^, ^31^ The study's explanatory variable of main interest was the health care region which the patient belonged to. We labeled these regions A, B, C, D, E and F, for anonymity.

For categorical variables Chi square test and Fischer's exact test was used to test significances. For the continuous version of the age variable ordinary ANOVA was used.

Survival time was calculated from diagnosis to death or until censoring. Patients were censored at end of and loss to follow‐up. Observed OS is presented using Kaplan Meier curves with log rank test. Log rank test is given for significance in the results text, if not otherwise conveyed by the context.

Univariate and multivariate analyses of variables considered associated with survival included age, ISS (international staging system) stage, gender, and time period of diagnosis. The whole cohort was investigated separately and in subgroups divided in patients receiving ASCT and patients not receiving ASCT. The latter group was analyzed divided in patients below 75 years of age at diagnosis and patients 75 years of age and older at diagnosis.

Multivariate analyses are presented with Hazard ratios (HR) using Cox proportional Hazard's regression model with 95% confidence intervals (CI). Region A was set as the reference region with HR 1 since that region had a superior survival in the total group MM treated patients.

*p* values < .05 were considered significant. *p* values were not adjusted for multiple testing and are presented for hypothesis generating purposes. Not all subgroup analyses are presented. Proportionality of hazards was assessed using Schoenfeld residuals, if *p* < .05 we assumed the HR to be an estimate of the average effect over time. Missing data was handled through complete case analysis.

Separate analyses were performed for patients alive 6 months after diagnosis to adjust for time to treatment bias regarding patients not eligible for treatment before that point in time. Separate multivariate analyses including treatment variables were also performed for hypothesis generating purposes.

Initial modern treatment was analyzed with regions grouped according to highest, intermediate, and low use in relation to OS. Region A is the region with highest use in all the comparisons. The regions included in the intermediate and low usage groups varies and were grouped depending on their approximately similar levels of percentages. The percentage cutoffs between the intermediate and low usage groups therefore also varies accordingly depending on the investigated subgroup. Regions included in each category for each subgroup are specified in connection with Figures 2A,B, S1 and S2. The specific percentage of usage in each respective region is presented in Tables 1 and S2–S4.

**TABLE 1:** Patient characteristics, all treated patients with multiple myeloma

		Region A	Region B	Region C	Region D	Region E	Region F	*p*
*n* = 5326		892	551	1175	968	1041	699	
Age (mean [SD])		68.66 (11.74)	70.71 (11.15)	70.51 (10.61)	70.86 (10.96)	70.85 (10.86)	69.99 (11.42)	<.001
Age (*n* [%])	0–49	58 (6.5)	18 (3.3)	39 (3.3)	35 (3.6)	48 (4.6)	39 (5.6)	.002
	50–59	123 (13.8)	72 (13.1)	134 (11.4)	110 (11.4)	102 (9.8)	81 (11.6)	
	60–69	274 (30.7)	153 (27.8)	342 (29.1)	272 (28.1)	276 (26.5)	193 (27.6)	
	70–79	264 (29.6)	179 (32.5)	412 (35.1)	315 (32.5)	377 (36.2)	225 (32.2)	
	80–	173 (19.4)	129 (23.4)	248 (21.1)	236 (24.4)	238 (22.9)	161 (23.0)	
Sex (*n* [%])	Female	364 (40.8)	231 (41.9)	488 (41.5)	415 (42.9)	450 (43.2)	306 (43.8)	.813
	Male	528 (59.2)	320 (58.1)	687 (58.5)	553 (57.1)	591 (56.8)	393 (56.2)	
Stage (*n* [%])	Stage I	148 (20.0)	116 (24.0)	167 (23.8)	124 (17.9)	130 (19.0)	120 (23.7)	<.001
	Stage II	388 (52.5)	185 (38.2)	316 (45.0)	280 (40.3)	280 (40.9)	229 (45.3)	
	Stage III	203 (27.5)	183 (37.8)	219 (31.2)	290 (41.8)	274 (40.1)	157 (31.0)	
	NA	153	67	473	274	357	193	
Modern treatm (*n* [%])^a^	No	303 (34.0)	265 (48.1)	629 (53.5)	535 (55.3)	533 (51.2)	358 (51.2)	<.001
	Yes	589 (66.0)	286 (51.9)	546 (46.5)	433 (44.7)	508 (48.8)	341 (48.8)	
HDT with ASCT (*n* [%])	No	551 (62.6)	369 (67.2)	815 (70.1)	705 (73.6)	758 (73.6)	475 (68.8)	<.001
	Yes	329 (37.4)	180 (32.8)	348 (29.9)	253 (26.4)	272 (26.4)	215 (31.2)	
	NA	12	2	12	10	11	9	
Consolid. ther. (*n* [%])	No	799 (92.1)	483 (88.6)	1038 (91.1)	808 (85.8)	908 (88.8)	571 (84.8)	<.001
	Yes	69 (7.9)	62 (11.4)	102 (8.9)	134 (14.2)	114 (11.2)	102 (15.2)	
	NA	24	6	35	26	19	26	
Within study (*n* [%])^b^	No	794 (93.4)	517 (96.3)	1090 (97.9)	835 (94.6)	950 (93.5)	644 (98.0)	<.001
	Yes	56 (6.6)	20 (3.7)	23 (2.1)	48 (5.4)	66 (6.5)	13 (2.0)	
	NA	42	14	62	85	25	42	
Alive 6 months (*n* [%])	FALSE	77 (8.6)	77 (14.0)	121 (10.3)	93 (9.6)	100 (9.6)	93 (13.3)	.003
	TRUE	815 (91.4)	474 (86.0)	1054 (89.7)	875 (90.4)	941 (90.4)	606 (86.7)	
Period of diagnosis (*n* [%])	<2012	357 (40.0)	216 (39.2)	440 (37.4)	360 (37.2)	374 (35.9)	276 (39.5)	.433
	≥2012	535 (60.0)	335 (60.8)	735 (62.6)	608 (62.8)	667 (64.1)	423 (60.5)	

a No is either no or missing.

b First line treatment in clinical study.

Abbreviation: ASCT, autologous stem cell transplantation; HDT, high dose chemotherapy.

Modern initial treatment was defined as bortezomib in combination with either melphalan, cyclophosphamide, thalidomide, or treatment with lenalidomide, pomalidomide, carfilzomib or daratumumab regardless of combination. In the data no discrimination could be done between no modern initial treatment and data missing. Consolidation/maintenance treatment was included as a separate variable. To adjust for differences in delay of reporting between the regions, only treatments started within a year from MM diagnosis were included. Later relapse treatment was not evaluated. Patients who did not receive treatment were excluded. Treatment is interpreted as intention to treat analyses in the study. R software was used for statistical analyses.^32^, ^33^

The study was approved by the ethical committee of Stockholm County (Dnr 2018/60, with approved amendment to Etikprövningsnämnden Dnr 2020‐00394).

## 3 RESULTS

Overall, in all six regions 5326 patients with symptomatic myeloma were analyzed after exclusion of 250 patients who did not receive treatment. The coverage for the Register during 2008–2017 compared to the Swedish cancer registry was 99.2% for the report at diagnosis. Coverage for the one‐year report compared to reported diagnosis of MM to the register was lower in region A (82.6%) compared to the rest of the regions (95.2%–99.2%). Age adjusted incidence for all symptomatic myeloma was with 95% CI: 6.3 (5.7–7.0), 6.1 (5.3–7.0), 6.0 (5.5–6.6), 5.9 (5.2–6.5), 6.4 (5.8–7.1) and 7.3 (6.4–8.2) per 100 000 for region A, B, C, D, E and F respectively. The proportion of smoldering myeloma was 18% overall, varying from 14% in region F to 23% in region B.

Age varied between the regions and patients were younger in Region A than in other regions (mean age 68.7 years for region A compared to 70.0–70.9 years for the rest). The distribution of gender did not differ significantly. ISS stage differed with highest percentage for stage I in region B and highest percentage for stage II in group A (Table 1).

The use of modern initial treatment, as defined above, differed between the regions (Tables 1 and S1). The highest percentage (66%) was seen in region A compared to other regions (45%–52%). The percentage of patients treated with ASCT also differed with highest percentage (37%) in region A compared to the rest (26%–33%). Consolidation/maintenance treatment was most frequent in region F (15%) and least frequent in region A (8%). In region A, 91% were alive 6 months after diagnosis compared to 86%–90% for the rest of the regions. The number of patients with first line treatment given within a clinical trial was most frequent in region A and E (7%) compared to the other regions (2%–5%). Patient characteristics in subgroups are provided in supplemental tables (Tables S2–S4).

### 3.1 Survival in all patients

**FIGURE 1:**
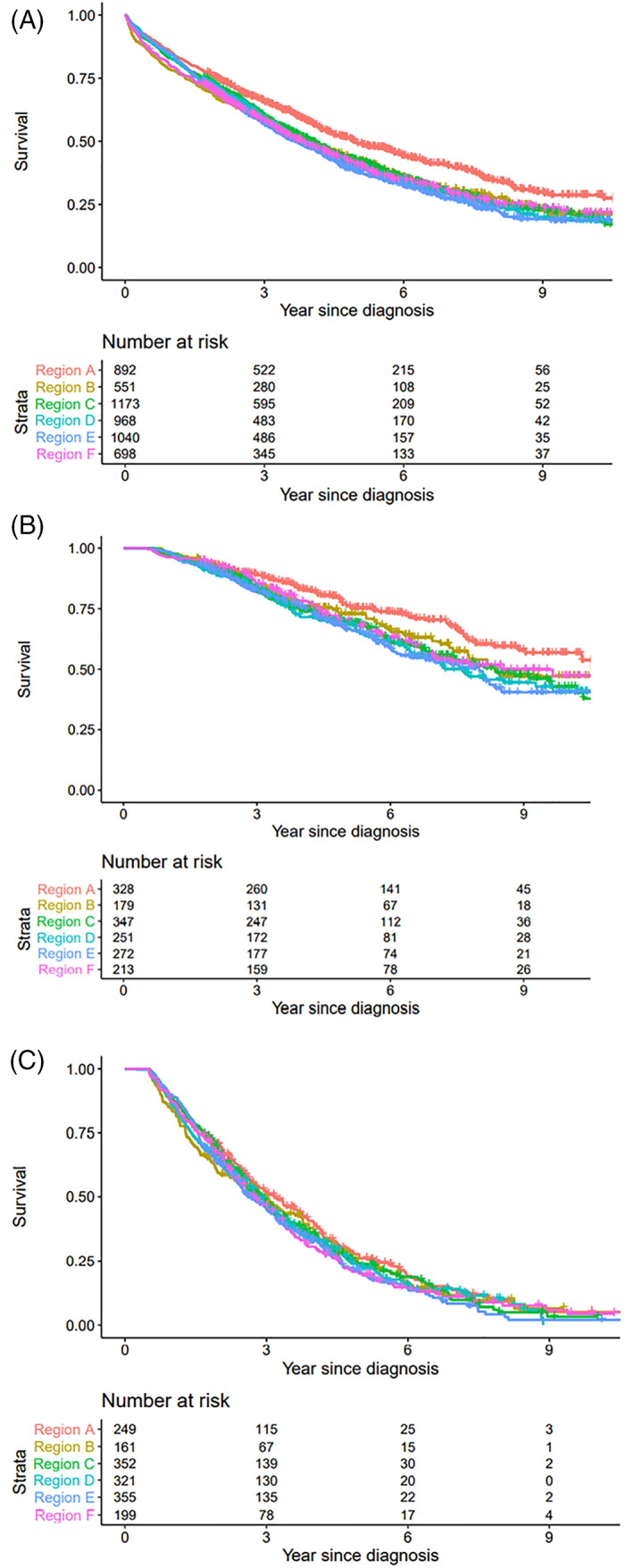
(A Overall observed survival by health care region, all treated patients, with number at risk. (B) Overall survival by health care region, patients treated with ASCT and alive 6 months after diagnosis, with number at risk. (C) Overall survival by health care region, patients not treated with ASCT, 75 years or older and alive 6 months after diagnosis, with number at risk

In the whole group of MM patients who received treatment, a significant superior OS for region A was observed when compared separately to region B, C, D, E and F (*p* < .01 for all regions respectively), (Figure 1A). This was true also when including only patients alive 6 months after diagnosis (*p* < .01 for all except *p* = .02 for region A vs. region B).

**FIGURE 2:**
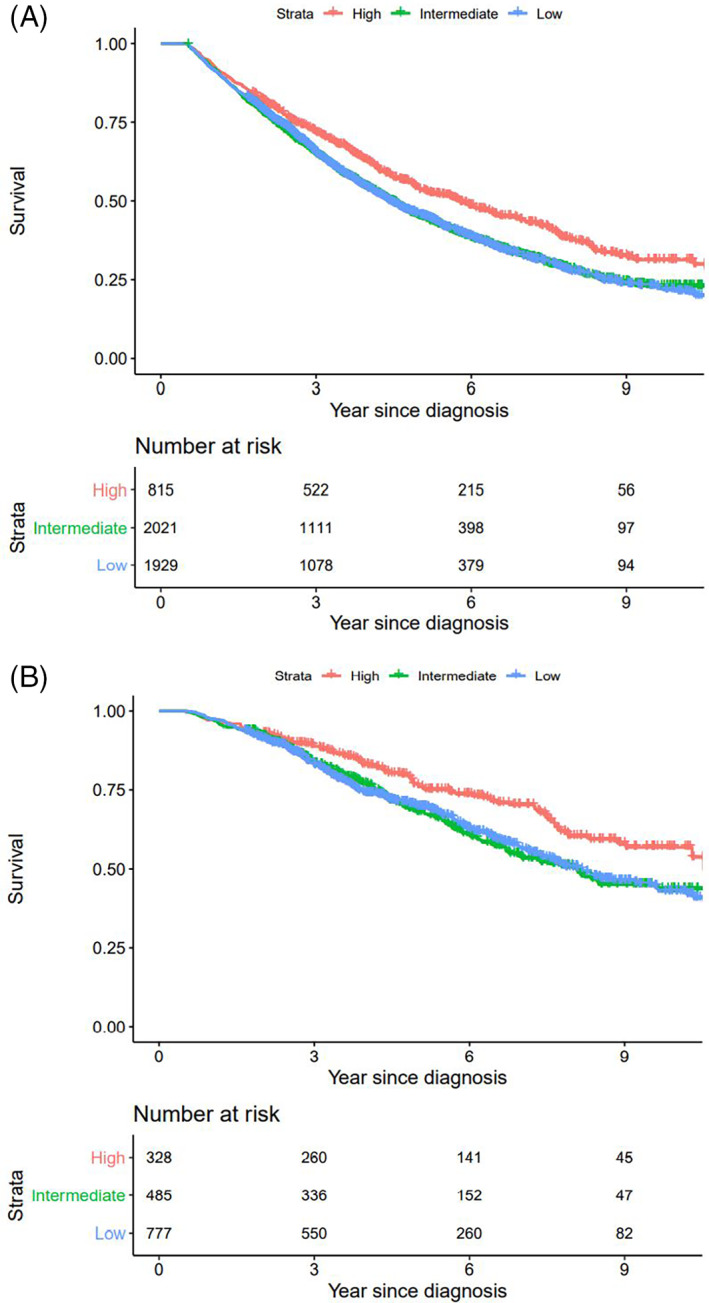
(A) Overall survival by regions with high, intermediate, and low usage of modern initial treatment, all treated patients alive 6 months after diagnosis, with number at risk. Region A (red), region B, E, F (green), region C, D (blue). (B) Overall survival by regions with high, intermediate, and low usage of modern initial treatment, patients treated with ASCT and alive 6 months after diagnosis, with number at risk. Region A (red), region E, F (green), region B, C, D (blue)

When all patients alive after 6 months were divided according to region and usage of modern initial therapy there was a superior survival for the region with highest use when compared to regions in the intermediate and low usage groups respectively, *p* < .01 for both, (Figure 2A).

**FIGURE 3:**
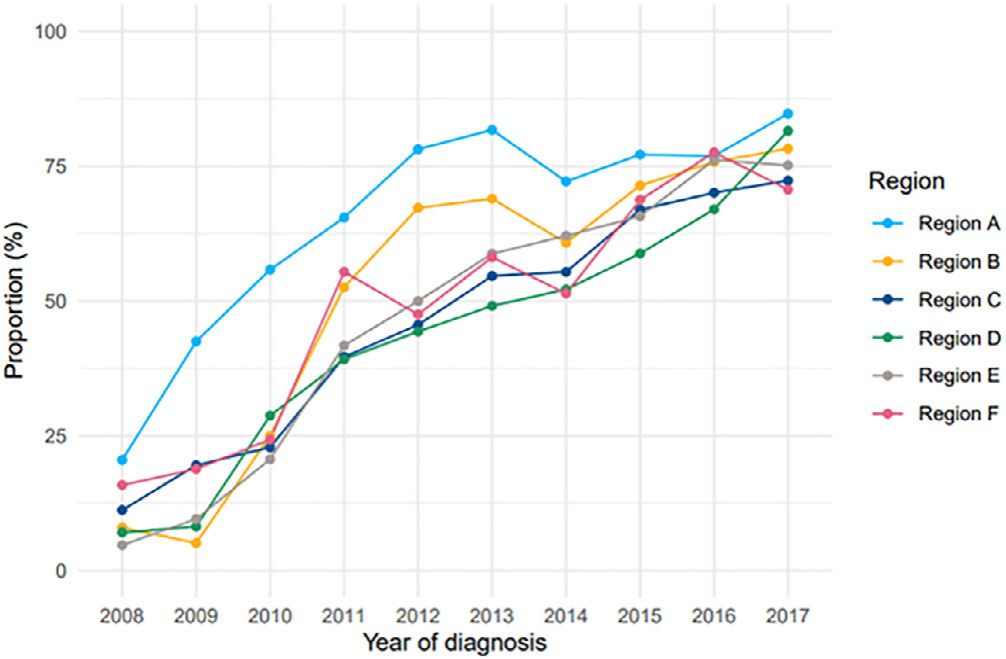
Proportion receiving initial modern treatment by region and year

Initial modern treatment appeared to increase in the regions over time (Figure 3).

### 3.2 Survival in ASCT patients

For patients receiving ASCT there was a significant superior survival in region A compared to region C, D, E and F, (*p* = .01, *p* < .01, *p* < .01, *p* = .04 respectively). The difference did not reach significance when comparing region, A and B (*p* = .08). A similar result was seen for region A versus B, C, D, E, F when including only patients alive 6 months after diagnosis (*p* = .08, *p* < .01, *p* < .01, *p* < .01, *p* = .05), (Figure 1B).

When performing univariate analysis in the patients alive 6 months after diagnosis, we observed that besides regions, the following variables were also associated with significantly superior survival: modern versus not modern initial treatment (*p* = .01), consolidation/maintenance treatment versus not (*p* = .03), ISS stage I versus II, III and stage missing (*p* < .01 for all respectively), time period of diagnosis ≥year 2012 versus <year 2012 (*p* = .01), age 0–49 years versus age 50–59, 60–69 and 70–79 (*p* = .02, *p* < .01, *p* < .01 respectively). Gender did not have an impact as a factor affecting OS (*p* = .96). Results were very similar when also including all patients receiving ASCT (Tables S5 and S6).

Multivariate analyses for patients alive after 6 months showed that a significant difference in survival remained for region A compared to C, D, E and F (Table 2). Test of proportional hazards was non‐significant (*p* = .14) for the global analysis but not for age (*p* = .01).

**TABLE 2:** Multivariate analysis, only patients alive 6 months after diagnosis

*n* complete cases		All (treated) patients		ASCT		No ASCT < 75		No ASCT > =75	
		4737		1590		1510		1637	
Variable		Hazard ratio	*p* value	Hazard ratio	*p* value	Hazard ratio	*p* value	Hazard ratio	*p* value
Region	A	1		1		1		1	
	B	1.09 (0.94–1.28)	.25	1.35 (0.99–1.86)	.06	1.04 (0.78–1.37)	0.8	1.04 (0.82–1.3)	.76
	C	1.1 (0.97–1.25)	.13	1.43 (1.09–1.86)	.01	1.04 (0.83–1.3)	0.71	0.97 (0.8–1.17)	.74
	D	1.13 (0.99–1.28)	.07	1.56 (1.18–2.06)	0	0.98 (0.78–1.23)	0.83	0.99 (0.81–1.2)	.88
	E	1.18 (1.03–1.34)	.01	1.56 (1.18–2.06)	0	1 (0.8–1.26)	0.98	1.05 (0.87–1.27)	.59
	F	1.13 (0.98–1.31)	.09	1.38 (1.03–1.86)	.03	1.06 (0.82–1.37)	0.67	1.04 (0.84–1.28)	.73
Stage	I	1		1		1		1	
	II	1.39 (1.22–1.59)	0	1.45 (1.15–1.82)	0	1.51 (1.2–1.91)	0	1.15 (0.9–1.46)	.27
	III	2.02 (1.76–2.32)	0	1.97 (1.53–2.53)	0	2.07 (1.63–2.64)	0	1.83 (1.43–2.33)	0
	Missing	1.84 (1.6–2.1)	0	1.57 (1.21–2.04)	0	1.83 (1.44–2.32)	0	1.75 (1.38–2.22)	0
Age	0–49	1		1		1		NA	NA
	50–59	1.32 (1.01–1.74)	.04	1.39 (1.03–1.87)	.03	0.79 (0.39–1.57)	0.5	NA	NA
	60–69	1.99 (1.55–2.55)	0	1.69 (1.27–2.24)	0	1.2 (0.66–2.19)	0.56	NA	NA
	70–79	3.2 (2.5–4.09)	0	2.3 (1.23–4.33)	.01	1.2 (0.66–2.18)	0.56	1	
	80–	6.53 (5.08–8.38)	0	NA	NA	NA	NA	1.73 (1.54–1.94)	0
Time period	<2012	1		1		1		1	
	≥2012	0.79 (0.73–0.86)	0	0.76 (0.64–0.91)	0	0.75 (0.65–0.86)	0	0.85 (0.75–0.95)	0

Abbreviation: ASCT, autologous stem cell transplantation.

When patients alive after 6 months were divided by region and use of modern initial therapy there was a superior survival for the group with highest use compared to regions in the intermediate and low usage groups respectively (*p* < .01 for both), (Figure 2B). This was not the case for the two subgroups not receiving ASCT (Figures S1 and S2).

### 3.3 Survival in non‐transplanted patients below 75 years of age

For these patients we did not see any significant difference in OS between region A and other regions. There was also no significant difference in OS when including only patients still alive 6 months after diagnosis (Figure S3).

Univariate analysis in patients alive after 6 months showed that the following variables were associated with superior survival: modern versus not modern initial treatment (*p* < .01), consolidation/maintenance treatment versus not (*p* = .02), ISS stage I versus II, III and stage missing (*p* < .01 for all respectively) and time period of diagnosis ≥2012 versus <2012 (*p* < .01). Age 0–49 years versus 50–59, 60–69 and 70–79 years of age was not significant (*p* = .36, *p* = .55, *p* = .50 respectively), as was also not gender (*p* = .55). In univariate analysis for all patients not receiving ASCT younger than 75 years of age, the results were very similar (Tables S5 and S6).

In multivariate analyses there were also no significant differences in survival between region A and the other regions (Table 2). Test of proportional hazards was non‐significant for the global analysis (*p* = .07) but not for age (*p* = .04).

### 3.4 Survival in non‐transplanted patients 75 years of age and older

In this group of patients, we noted a superior OS for region A compared to region B, E and F (*p* = .02, *p* = .04, *p* = .02 respectively). When including only patients alive 6 months after diagnosis, a significant superior difference in survival could however possibly only be noted between region A and E (log rank *p* = .04, HR 1.2, CI 1.00–1.44, *p* = .06), (Figure 1C).

Univariate analysis in patients alive after 6 months revealed these variables to be associated to superior survival: modern versus not modern initial treatment (*p* < .01), consolidation/maintenance treatment versus not (*p* = .01), ISS stage I versus III and versus stage missing (*p* < .01 and *p* < .01 respectively). Age 70–79 years versus 80 years or older (*p* < .01) and time period of diagnosis ≥2012 versus <2012 (*p* < .01). Gender was not significant (*p* = .46). In univariate analysis for all patients not receiving ASCT, 75 years and older, results were very similar (Tables S5 and S6).

In multivariate analysis no significant difference in OS between region A and the other regions remained (Table 2) Test of proportional hazards was non‐significant for the global analysis (*p* = .76).

Multivariate analyses including the variables modern initial treatment and consolidation/maintenance treatment were also performed. These factors appeared significant for superior survival for the two subgroups of patients not treated with ASCT. In patients treated with ASCT however, the variables modern initial treatment and no consolidation/maintenance treatment did not turn out as significant (Tables S7–S9).

## 4 DISCUSSION

In this nationwide study, using population‐based data from 5326 patients with symptomatic myeloma in the Swedish Myeloma Register, we could demonstrate an improved survival over time and identify age at diagnosis, ISS stage but also use of novel drugs as initial treatment and consolidation/maintenance treatment as variables associated with improved survival. Importantly we also found regional differences. In the total group of MM patients, a superior OS was observed in region A when compared to all other regions separately. The difference was evident in patients undergoing ASCT where region A had a significantly better OS compared to all regions except one, while in patients not receiving ASCT and 75 years or older, the difference in survival was no longer evident after adjusting for a possible time to treatment bias of 6 months.

The strength of this study is that it represents unselected and comprehensive data from the whole Swedish population including all age categories and stages for comparisons during a long period of time. Limitations include coverage for the one‐year report which was less frequent in region A than in other regions, thus excluding more patients in that region from analyses in our study. This could possibly have biased the survival results and perhaps also the age distribution in that region. The fact that our analyses do not include data on comorbidity is also a limitation possibly implicating residual confounding. The results in univariate and multivariate analyses must also be interpreted with consideration for the limitations of a retrospective study.

Adapting modern initial treatment methods (new agents and the combination of these) was significant for superior survival in univariate analysis in all the subgroups. Use of modern initial treatment differed between the regions and was most common in region A. When categorizing regions into low, intermediate and high use of modern initial treatment, the region with highest use (region A) had a superior survival in the whole group of patients and in the subgroup with patients receiving ASCT. Usage appeared to be more frequent in region A in earlier years but seemed to even out between the regions over time. Since time period of diagnosis was significant for superior survival in all subgroups in general, it appears likely that use of modern initial treatment in the regions, time period of diagnosis and survival were closely linked. It is therefore possible that the differences in survival between the regions were most pronounced during the early time period of diagnosis.

Possible residual regional confounding factors in region A such as different socioeconomic circumstances, education, infrastructure, and access to healthcare centers may also have been important for better survival although we could not assess these factors further in the study. However, with the combination of high use of modern initial treatment and superior survival for ASCT treated patients in region A, it seems that survival for ASCT treated patients may possibly have depended on modern initial treatment. The reasons for the choice of treatment and the connection to other factors however remain a matter of speculation. It is perhaps surprising that the survival difference is to be found in the ASCT group and not in the other two subgroups since ASCT patients possibly receive a more standardized treatment regimen. However, in the other subgroups there is likely also a higher risk of general comorbidity and death from other causes than myeloma, making it more difficult to relate long term differences in survival specifically to treatment. In this study, for the subgroups not receiving ASCT, we could not show that differences in usage of modern initial treatment (highest vs. intermediate and low) also translated into a significant difference in OS.

Of note is that modern initial treatment, when included in multivariate analysis with consideration for a time‐to‐treatment bias, could not adjust for the difference in survival between the regions in patients treated with ASCT. However, this approach to evaluate treatment may perhaps be considered imprecise and speculative in nature. Also, one must consider that the number of cycles of modern initial treatment before ASCT may have varied between the regions possibly affecting both response and survival.

In patients not receiving ASCT, consolidation/maintenance treatment appeared significant for superior survival in both univariate and multivariate analysis in both subgroups. Considering the usage pattern and low reported usage overall, it is however not likely that this factor explained differences in survival between region A and the other health care regions.

For all treated MM patients there seemed to be large variations in ISS stage between regions and a major factor for these variations was the missing assessment of ISS. The survival for patients with non‐evaluated ISS stage in the whole group appeared to resemble survival for ISS stage III. It therefore seems likely to assume that most patients with ISS stage missing also had a disease with poor prognosis. Possibly these patients were not tested for Beta2 microglobulin (part of the ISS evaluation) routinely at diagnosis in some regions and could for this reason not be further classified.

There were also differences in age between the regions. In the whole study group, percentage of patients with age 0–49 years, 50–59 years and 60–69 years were highest in region A. In patients receiving ASCT however, the age group 0–49 years, had highest percentage in region E, F and A. Although age was significant for survival in multivariate analysis in this group of patients, the differences in OS between region A and the other regions remained. Our conclusion is therefore that age alone was not sufficient to explain survival differences between the regions.

In conclusion we observed a better survival in region A for patients receiving ASCT compared to other regions. A higher usage of initial modern treatment or regional residual confounding may provide explanations. In patients not receiving ASCT, 75 years or older, survival differences were not evident after considering a time to treatment bias of 6 months.

## CONFLICT OF INTEREST

The authors have stated explicitly that there are no conflicts of interest in connection with this article.

## AUTHOR CONTRIBUTIONS

**Göran Wålinder:** Conceptualization (equal); formal analysis (equal); methodology (equal); resources (equal); visualization (equal); writing – original draft (lead); writing – review and editing (equal). **Anna Genell:** Formal analysis (equal); methodology (equal); resources (equal); visualization (equal); writing – review and editing (equal). **Gunnar Juliusson:** Resources (equal); writing – review and editing (equal). **Ronald Svensson:** Resources (equal); writing – review and editing (equal). **Antonio Izarra Santamaria:** Resources (equal); writing – review and editing (equal). **Jacob Crafoord:** Resources (equal); writing – review and editing (equal). **Kristina Carlson:** Resources (equal); writing – review and editing (equal). **Dorota Knut‐Bojanowska:** Resources (equal); writing – review and editing (equal). **Ljupco Veskovski:** Resources (equal); writing – review and editing (equal). **Birgitta Lauri:** Resources (equal); writing – review and editing (equal). **Johan Lund:** Resources (equal); writing – review and editing (equal). **Ingemar Turesson:** Resources (equal); writing – original draft (supporting); writing – review and editing (equal). **Markus Hansson:** Resources (equal); writing – review and editing (equal). **Cecilie Hveding Blimark:** Resources (equal); writing – review and editing (equal). **Hareth Nahi:** Conceptualization (equal); formal analysis (equal); funding acquisition (lead); methodology (equal); project administration (equal); resources (equal); supervision (lead); visualization (equal); writing – original draft (supporting); writing – review and editing (equal).

## ETHICS STATEMENT

The study was approved by the ethical committee of Stockholm County (Dnr 2018/60, with approved amendment to Etikprövningsnämnden Dnr 2020–00394) and in line with the declaration of Helsinki. Before a patient is registered in The Swedish Myeloma Register, they must be informed about registration and that data can be used for research. The health care provider reporting to the register is responsible for giving this information to each patient.

## Supporting information


**Appendix S1**: Supporting Information

## Data Availability

Data can be shared upon request to the corresponding author, in accordance with ethical and privacy restrictions and the procedures of Karolinska University hospital.
