# Supplementary material for: Regional differences in treatment and outcome for myeloma patients in Sweden: A population based Swedish myeloma register study
Source: Cancer Rep (Hoboken). 2022 Mar 3;5(11):e1614. doi: 10.1002/cnr2.1614 (PMC9675390; doi:10.1002/cnr2.1614)

**Supplemental table 1. Modern initial treatment for all treated patients by age and region**

**Supplemental table 2. Patient characteristics, patients treated with ASCT.**

|  |  | **Region A** | **Region B** | **Region C** | **Region D** | **Region E** | **Region F** | **p** |
| --- | --- | --- | --- | --- | --- | --- | --- | --- |
| n = 1597 |  | 329 | 180 | 348 | 253 | 272 | 215 |  |
| Age (mean (SD)) |  | 58.09 (8.38) | 59.57 (6.95) | 59.34 (7.06) | 57.57 (7.43) | 57.97 (7.58) | 58.51 (8.28) | 0.018 |
| Age (n (%)) | 0-49 | 51 (15.5) | 16 (8.9) | 35 (10.1) | 33 (13.0) | 43 (15.8) | 34 (15.8) | 0.038 |
|  | 50-59 | 104 (31.6) | 63 (35.0) | 112 (32.2) | 100 (39.5) | 85 (31.2) | 64 (29.8) |  |
|  | 60-69 | 171 (52.0) | 95 (52.8) | 191 (54.9) | 119 (47.0) | 141 (51.8) | 111 (51.6) |  |
|  | 70-79 | 3 (0.9) | 6 (3.3) | 10 (2.9) | 1 (0.4) | 3 (1.1) | 6 (2.8) |  |
| Sex (n (%)) | Female | 124 (37.7) | 56 (31.1) | 124 (35.6) | 91 (36.0) | 110 (40.4) | 82 (38.1) | 0.465 |
|  | Male | 205 (62.3) | 124 (68.9) | 224 (64.4) | 162 (64.0) | 162 (59.6) | 133 (61.9) |  |
| Stage (n (%)) | Stage I | 72 (24.7) | 70 (42.2) | 92 (38.3) | 64 (29.9) | 68 (28.8) | 61 (33.5) | 0.002 |
|  | Stage II | 156 (53.4) | 56 (33.7) | 100 (41.7) | 92 (43.0) | 108 (45.8) | 84 (46.2) |  |
|  | Stage III | 64 (21.9) | 40 (24.1) | 48 (20.0) | 58 (27.1) | 60 (25.4) | 37 (20.3) |  |
|  | NA | 37 | 14 | 108 | 39 | 36 | 33 |  |
| Modern Treatm (n (%)) * | No | 48 (14.6) | 45 (25.0) | 86 (24.7) | 66 (26.1) | 46 (16.9) | 36 (16.7) | 0.001 |
|  | Yes | 281 (85.4) | 135 (75.0) | 262 (75.3) | 187 (73.9) | 226 (83.1) | 179 (83.3) |  |
| Consolid. ther. (n (%)) | No | 283 (86.3) | 161 (89.4) | 326 (93.9) | 209 (82.9) | 236 (87.1) | 173 (81.2) | <0.001 |
|  | Yes | 45 (13.7) | 19 (10.6) | 21 (6.1) | 43 (17.1) | 35 (12.9) | 40 (18.8) |  |
|  | NA | 1 | 0 | 1 | 1 | 1 | 2 |  |
| Within study (n (%)) ** | No | 307 (95.3) | 174 (97.8) | 323 (97.0) | 206 (92.8) | 260 (95.6) | 203 (98.1) | 0.050 |
|  | Yes | 15 (4.7) | 4 (2.2) | 10 (3.0) | 16 (7.2) | 12 (4.4) | 4 (1.9) |  |
|  | NA | 7 | 2 | 15 | 31 | 0 | 8 |  |
| Alive 6 months (n (%)) | FALSE | 1 (0.3) | 1 (0.6) | 1 (0.3) | 2 (0.8) | 0 (0.0) | 2 (0.9) | 0.626 |
|  | TRUE | 328 (99.7) | 179 (99.4) | 347 (99.7) | 251 (99.2) | 272 (100.0) | 213 (99.1) |  |
| Period of Diagnosis (n (%)) | <2012 | 135 (41.0) | 69 (38.3) | 137 (39.4) | 103 (40.7) | 95 (34.9) | 89 (41.4) | 0.658 |
|  | >=2012 | 194 (59.0) | 111 (61.7) | 211 (60.6) | 150 (59.3) | 177 (65.1) | 126 (58.6) |  |

*No is either no or missing.

**First line treatment in clinical study

**Supplemental table 3. Patient characteristics, patients not treated with ASCT below 75 years of age.**

|  |  | **Region A** | **Region B** | **Region C** | **Region D** | **Region E** | **Region F** | **p** |
| --- | --- | --- | --- | --- | --- | --- | --- | --- |
| n = 1674 |  | 261 | 153 | 382 | 324 | 337 | 217 |  |
| Age (mean (SD)) | | 68.05 (6.23) | 68.31 (5.85) | 68.46 (5.19) | 68.84 (4.51) | 68.61 (5.41) | 68.12 (5.96) | 0.510 |
| Age (n (%)) | 0-49 | 6 (2.3) | 2 (1.3) | 4 (1.0) | 2 (0.6) | 5 (1.5) | 4 (1.8) | 0.447 |
|  | 50-59 | 19 (7.3) | 9 (5.9) | 21 (5.5) | 10 (3.1) | 17 (5.0) | 15 (6.9) |  |
|  | 60-69 | 100 (38.3) | 57 (37.3) | 151 (39.5) | 150 (46.3) | 133 (39.5) | 79 (36.4) |  |
|  | 70-79 | 136 (52.1) | 85 (55.6) | 206 (53.9) | 162 (50.0) | 182 (54.0) | 119 (54.8) |  |
| Sex (n (%)) | Female | 107 (41.0) | 63 (41.2) | 155 (40.6) | 138 (42.6) | 134 (39.8) | 92 (42.4) | 0.981 |
|  | Male | 154 (59.0) | 90 (58.8) | 227 (59.4) | 186 (57.4) | 203 (60.2) | 125 (57.6) |  |
| Stage (n (%)) | Stage I | 44 (20.1) | 25 (18.8) | 50 (19.8) | 36 (15.7) | 39 (17.3) | 34 (22.2) | <0.001 |
|  | Stage II | 118 (53.9) | 48 (36.1) | 126 (49.8) | 99 (43.0) | 82 (36.3) | 72 (47.1) |  |
|  | Stage III | 57 (26.0) | 60 (45.1) | 77 (30.4) | 95 (41.3) | 105 (46.5) | 47 (30.7) |  |
|  | NA | 42 | 20 | 129 | 94 | 111 | 64 |  |
| Modern Treatm (n (%)) * | No | 90 (34.5) | 71 (46.4) | 196 (51.3) | 171 (52.8) | 168 (49.9) | 110 (50.7) | <0.001 |
|  | Yes | 171 (65.5) | 82 (53.6) | 186 (48.7) | 153 (47.2) | 169 (50.1) | 107 (49.3) |  |
| Consolid. ther. (n (%)) | No | 240 (93.8) | 127 (85.2) | 325 (86.9) | 273 (85.0) | 286 (85.4) | 171 (81.4) | 0.004 |
|  | Yes | 16 (6.2) | 22 (14.8) | 49 (13.1) | 48 (15.0) | 49 (14.6) | 39 (18.6) |  |
|  | NA | 5 | 4 | 8 | 3 | 2 | 7 |  |
| Within study (n (%)) ** | No | 224 (90.0) | 141 (93.4) | 351 (97.2) | 283 (93.1) | 300 (90.1) | 197 (97.0) | <0.001 |
|  | Yes | 25 (10.0) | 10 (6.6) | 10 (2.8) | 21 (6.9) | 33 (9.9) | 6 (3.0) |  |
|  | NA | 12 | 2 | 21 | 20 | 4 | 14 |  |
| Alive 6 months (n (%)) | FALSE | 28 (10.7) | 20 (13.1) | 33 (8.6) | 24 (7.4) | 31 (9.2) | 28 (12.9) | 0.200 |
|  | TRUE | 233 (89.3) | 133 (86.9) | 349 (91.4) | 300 (92.6) | 306 (90.8) | 189 (87.1) |  |
| Period of Diagnosis (n (%)) | <2012 | 115 (44.1) | 55 (35.9) | 135 (35.3) | 125 (38.6) | 110 (32.6) | 79 (36.4) | 0.100 |
|  | >=2012 | 146 (55.9) | 98 (64.1) | 247 (64.7) | 199 (61.4) | 227 (67.4) | 138 (63.6) |  |

*No is either no or missing.

**First line treatment in clinical study

**Supplemental table 4. Patient characteristics, patients not treated with ASCT, 75 years or older.**

|  |  | **Region A** | **Region B** | **Region C** | **Region D** | **Region E** | **Region F** | **p** |
| --- | --- | --- | --- | --- | --- | --- | --- | --- |
| n = 1999 |  | 290 | 216 | 433 | 381 | 421 | 258 |  |
| Age (mean (SD)) |  | 80.96 (4.56) | 81.71 (4.84) | 81.05 (4.69) | 81.22 (4.49) | 80.76 (4.26) | 81.22 (4.53) | 0.215 |
| Age (n (%)) | 70-79 | 123 (42.4) | 87 (40.3) | 193 (44.6) | 149 (39.1) | 189 (44.9) | 100 (38.8) | 0.378 |
|  | 80- | 167 (57.6) | 129 (59.7) | 240 (55.4) | 232 (60.9) | 232 (55.1) | 158 (61.2) |  |
| Sex (n (%)) | Female | 129 (44.5) | 111 (51.4) | 201 (46.4) | 183 (48.0) | 199 (47.3) | 128 (49.6) | 0.688 |
|  | Male | 161 (55.5) | 105 (48.6) | 232 (53.6) | 198 (52.0) | 222 (52.7) | 130 (50.4) |  |
| Stage (n (%)) | Stage I | 31 (14.2) | 20 (10.9) | 25 (12.1) | 24 (9.8) | 21 (9.6) | 24 (14.5) | 0.013 |
|  | Stage II | 112 (51.1) | 81 (44.0) | 89 (43.2) | 87 (35.5) | 89 (40.8) | 72 (43.6) |  |
|  | Stage III | 76 (34.7) | 83 (45.1) | 92 (44.7) | 134 (54.7) | 108 (49.5) | 69 (41.8) |  |
|  | NA | 71 | 32 | 227 | 136 | 203 | 93 |  |
| Modern Treatm (n (%)) * | No | 154 (53.1) | 147 (68.1) | 335 (77.4) | 289 (75.9) | 308 (73.2) | 203 (78.7) | <0.001 |
|  | Yes | 136 (46.9) | 69 (31.9) | 98 (22.6) | 92 (24.1) | 113 (26.8) | 55 (21.3) |  |
| Consolid. ther. (n (%)) | No | 276 (97.2) | 194 (90.2) | 385 (92.3) | 326 (88.3) | 386 (92.8) | 227 (90.8) | 0.002 |
|  | Yes | 8 (2.8) | 21 (9.8) | 32 (7.7) | 43 (11.7) | 30 (7.2) | 23 (9.2) |  |
|  | NA | 6 | 1 | 16 | 12 | 5 | 8 |  |
| Within study (n (%)) ** | No | 263 (94.3) | 201 (97.1) | 413 (99.3) | 346 (96.9) | 390 (94.9) | 244 (98.8) | <0.001 |
|  | Yes | 16 (5.7) | 6 (2.9) | 3 (0.7) | 11 (3.1) | 21 (5.1) | 3 (1.2) |  |
|  | NA | 11 | 9 | 17 | 24 | 10 | 11 |  |
| Alive 6 months (n (%)) | FALSE | 41 (14.1) | 55 (25.5) | 81 (18.7) | 60 (15.7) | 66 (15.7) | 59 (22.9) | 0.003 |
|  | TRUE | 249 (85.9) | 161 (74.5) | 352 (81.3) | 321 (84.3) | 355 (84.3) | 199 (77.1) |  |
| Period of Diagnosis (n (%)) | <2012 | 106 (36.6) | 92 (42.6) | 164 (37.9) | 131 (34.4) | 168 (39.9) | 108 (41.9) | 0.272 |
|  | >=2012 | 184 (63.4) | 124 (57.4) | 269 (62.1) | 250 (65.6) | 253 (60.1) | 150 (58.1) |  |

*No is either no or missing.

**First line treatment in clinical study

**Supplemental table 5: Univariate analysis, only patients alive 6 months after diagnosis**

**Supplemental table 6. Univariate analyses, all treated patients.**

**Supplemental table 7. Multivariate for patients receiving ASCT and alive after 6 months**, **including initial modern treatment and consolidation treatment as variables.**


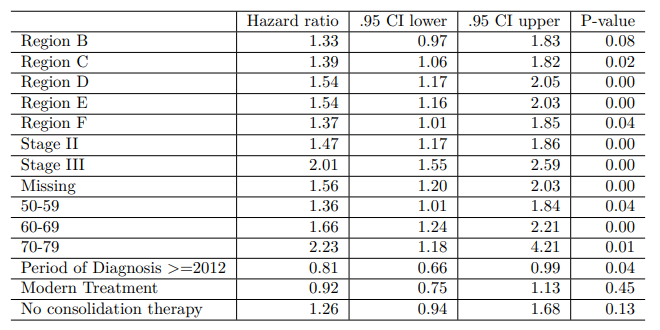


**Supplemental table 8. Multivariate for patients not receiving ASCT below 75 years of age and alive 6 months after diagnosis, including initial modern treatment and consolidation treatment as variables.**


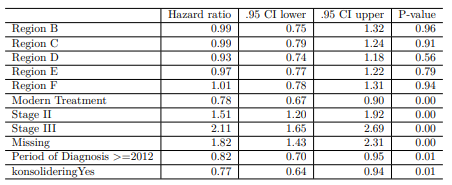


**Supplemental table 9. Multivariate analysis for patients not receiving ASCT 75 years of age or older and alive 6 months after diagnosis, including initial modern treatment and consolidation treatment as variables.**


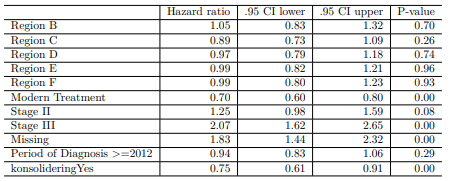


**Supplemental Figure 1. Overall survival by health care region and use of modern initial treatment, patients not treated with ASCT, below 75 years of age and alive 6 months after diagnosis, with number at risk. Region A (red), region B, E, F (green), region C, D (blue).**


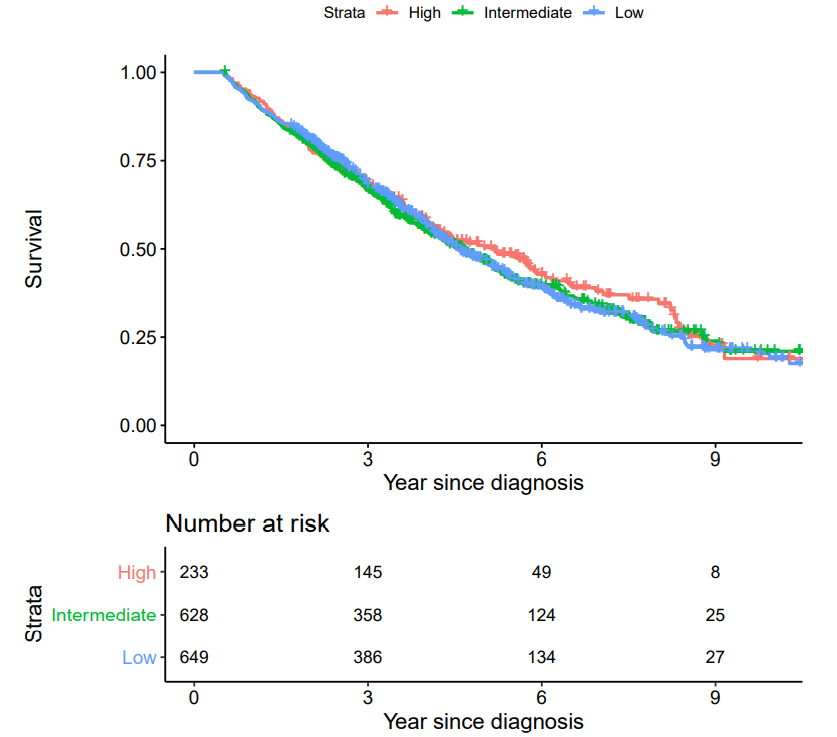


**Supplemental Figure 2. Overall survival by health care region and use of modern initial treatment. Patients not treated with ASCT, 75 years of age or older and alive 6 months after diagnosis, with number at risk. Region A (red), region B, E (green), region C, D, F (blue).**


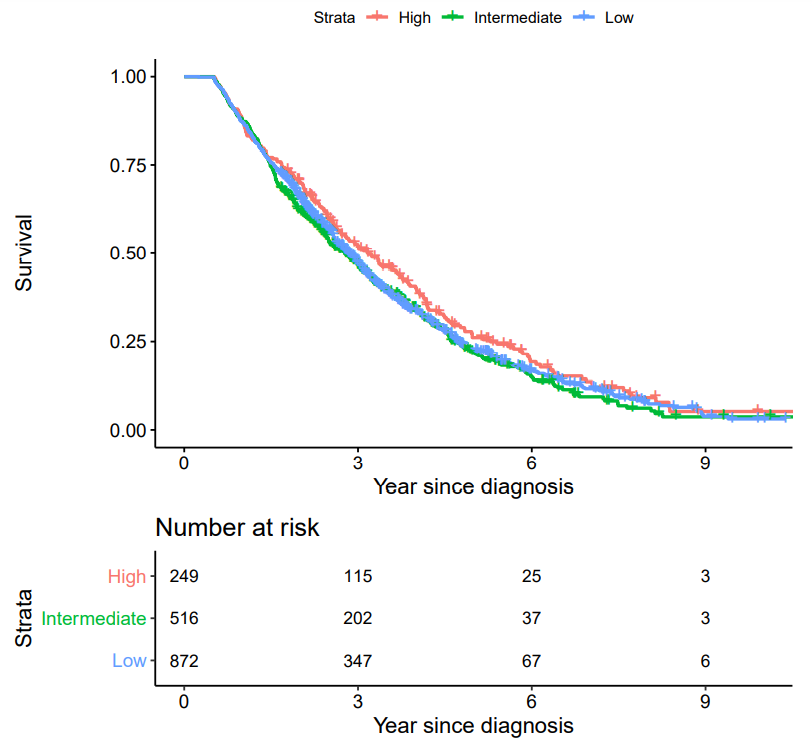


**Supplemental Figure 3. Overall survival by health care region, patients not treated with ASCT, below 75 years of age and alive 6 months after diagnosis, with number at risk.**


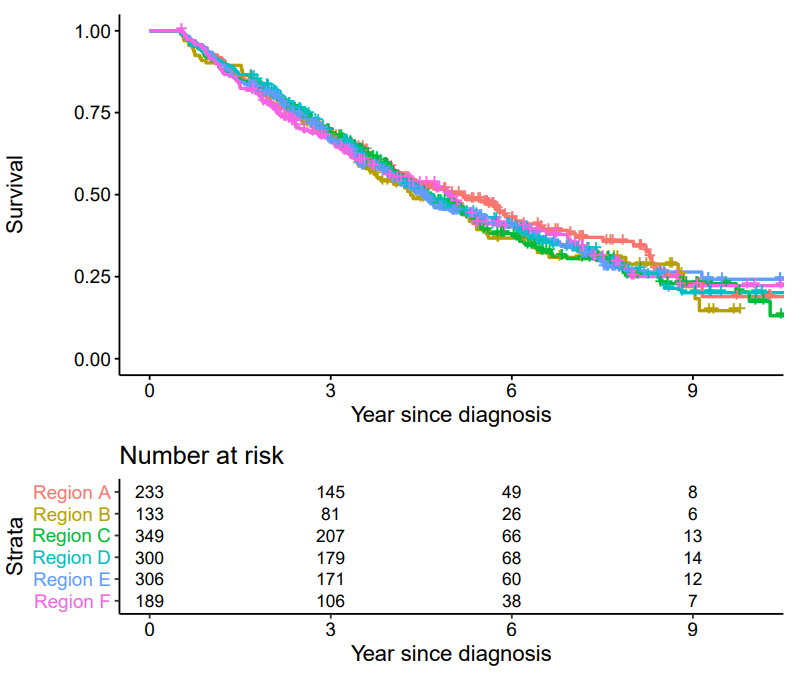

Supplement: Supplementary file 1 — Appendix S1: Supporting Information [file CNR2-5-e1614-s001.docx]
